# King Edward VII.'s Hospital Fund for London

**Published:** 1907-03-30

**Authors:** 


### King Edward VII.'s Hospital Fund for London.

The King's Hospital Fund has shown increasing vitality during each of the ten years of its existence. Its income last year from all sources amounted to upwards of £130,000, and its invested funds exceed one million sterling. It is a principle of sound hospital finance to create as many sources of income as possible. Three such sources of permanent revenue were started in the first year of the Fund's existence, and it was hoped that each source would ultimately produce at least £50,000 per annum. They were:—(1) Subscriptions and donations from the general public, which amounted to £29,903 in 1906; (2) revenue from investments and legacies, which amounted to £61,902 in the same year; and (3) subscriptions of one shilling and upwards through the League of Mercy, which yielded £18,000, an increase of £3,000 over the previous year. £111,000 was awarded to hospitals and convalescent homes in 1906, the largest amount distributed in any year since the Fund was instituted in 1897. The total sum thus distributed by the Fund amounts to nearly three-quarters of a million sterling. To quote such figures as these as the result of ten years' work is to prove the phenomenal success of the King's Fund.

The King's Fund has, however, accomplished many excellent things in other directions. It has actively promoted the amalgamation of groups of smaller hospitals devoted to the treatment of special diseases. Already it has secured the amalgamation of the orthopædic hospitals, and there is reason to hope that other groups of special hospitals may be amalgamated shortly. It has protected the supporters of voluntary hospitals from any diversion of their funds from the direct relief of the sick to the maintenance of medical schools. It has issued annually a statistical report on hospital expenditure and prices since 1904, with the result that the sixteen large general hospitals first included showed a considerable reduction in expenditure in the ensuing year, the value of which, taking into consideration the increase in the number of beds, is equal to a saving of about £20,000. Economy committees have been set up by a number of other hospitals which will no doubt take advantage of the information which these statistical reports contain, so that a still further and larger saving in expenditure may be expected in the near future.

It is a commonplace for certain relatively thoughtless people to declare that the effect of the King's Fund upon hospital finance has been harmful, in the sense that people, who formerly subscribed direct to individual hospitals, now divert their subscriptions to the King's Fund. We have made the most careful investigation of these and similar statements, and we can find no evidence whatever in the audited accounts of the hospitals to support this contention. Indeed, it is not a little remarkable that the very sources of the King's Fund revenue which, if successful, might have had the effect claimed—*i.e.* subscriptions and donations, are the only items which do not display a progressive increase year by year. Thus the amount received in annual subscriptions in 1906 was only £23,218, which is the lowest sum received from this source in any year, except the first, since the foundation of the Fund. In the same way the donations have yielded a less sum in 1906 than in any former year. The reason for this, we take it to be, is that no special effort has been made by the Fund to raise money from these sources, owing, no doubt, to a desire not to compete with the hospitals. We feel, however, that if the great middle-class public were to realise that they alone are the people who have yet to contribute regularly to the King's Fund, the £50,000 which these sources ought to yield would be speedily forthcoming. Another statement which the less thoughtful of hospital people sometimes make is that the King's Fund is now getting all the legacies. It is true that the Fund has received a few very handsome legacies from its deceased subscribers, but the list of legacies received during each year, as published on pp. 52 and 53 of the report, eloquently disproves the statement we have just mentioned.

The truth is that voluntary hospitals owe more, probably, to the King's Fund, than to any other agency which has been established in the world's history, in aid of the hospitals. No one who has any regard for his own reputation will venture to question the fact. This result is largely due to the great interest taken in the Fund by King Edward VII. and the Prince of Wales, who have each rendered, ungrudgingly, zealous personal service, to which circumstance the great success of the King's Fund is mainly due.

